# Effectiveness and cost-effectiveness of knowledge transfer and behavior modification interventions in type 2 diabetes mellitus patients—the INDICA study: a cluster randomized controlled trial

**DOI:** 10.1186/s13012-015-0233-1

**Published:** 2015-04-09

**Authors:** Yolanda Ramallo-Fariña, Lidia García-Pérez, Iván Castilla-Rodríguez, Lilisbeth Perestelo-Pérez, Ana María Wägner, Pedro de Pablos-Velasco, Armando Carrillo Domínguez, Mauro Boronat Cortés, Laura Vallejo-Torres, Marcos Estupiñán Ramírez, Pablo Pedrianes Martín, Ignacio García-Puente, Miguel Ángel Salinero-Fort, Pedro Guillermo Serrano-Aguilar, INDICA team

**Affiliations:** Fundación Canaria de Investigación Sanitaria (FUNCANIS), Tenerife, Spain; Servicio de Evaluación del Servicio Canario de la Salud (SESCS), Tenerife, Spain; Red de Investigación en Servicios de Salud en Enfermedades Crónicas (REDISSEC), Madrid, Spain; Centro de Investigaciones Biomédicas de Canarias (CIBICAN), Tenerife, Spain; Dpto de endocrinología, Complejo Hospitalario Universitario Insular Materno-Infantil, Gran Canaria, Spain; Instituto de Investigaciones Biomédicas y Sanitarias, Universidad de Las Palmas de Gran Canaria, Gran Canaria, Spain; Dpto de endocrinología, Hospital Universitario Dr. Negrín, Gran Canaria, Gran Canaria, Spain; Dpto de Economía de las Instituciones, Estadística Económica y Econometría, Universidad de la Laguna, Tenerife, Spain; Dpto de Ciencias Médicas y Quirúrgicas, Universidad de Las Palmas de Gran Canaria, Gran Canaria, Spain; Programas asistenciales, Servicio Canario de la Salud, Gran Canaria, Spain; Gerencia Adjunta de Planificación y Calidad. Servicio Madrileño de Salud (SERMAS), Madrid, Spain

**Keywords:** Behavior modification, Care management, Decision support aids, Electronic communication, Knowledge transfer, Mobile phone technology, Multicomponent intervention, Primary care, Type 2 diabetes mellitus

## Abstract

**Background:**

Type 2 diabetes mellitus is a chronic disease whose health outcomes are related to patients and healthcare professionals’ decision-making. The Diabetes Intervention study in the Canary Islands (INDICA study) aims to evaluate the effectiveness and cost-effectiveness of educational interventions supported by new technology decision tools for type 2 diabetes patients and primary care professionals in the Canary Islands.

**Methods/design:**

The INDICA study is an open, community-based, multicenter, clinical controlled trial with random allocation by clusters to one of three interventions or to usual care. The setting is primary care where physicians and nurses are invited to participate. Patients with diabetes diagnosis, 18–65 years of age, and regular users of mobile phone were randomly selected. Patients with severe comorbidities were excluded. The clusters are primary healthcare practices with enough professionals and available places to provide the intervention. The calculated sample size was 2,300 patients.

Patients in group 1 are receiving an educational group program of eight sessions every 3 months led by trained nurses and monitored by means of logs and a web-based platform and tailored semi-automated SMS for continuous support. Primary care professionals in group 2 are receiving a short educational program to update their diabetes knowledge, which includes a decision support tool embedded into the electronic clinical record and a monthly feedback report of patients’ results. Group 3 is receiving a combination of the interventions for patients and professionals.

The primary endpoint is the change in HbA1c in 2 years. Secondary endpoints are cardiovascular risk factors, macrovascular and microvascular diabetes complications, quality of life, psychological outcomes, diabetes knowledge, and healthcare utilization. Data is being collected from interviews, questionnaires, clinical examinations, and records. Generalized linear mixed models with repeated time measurements will be used to analyze changes in outcomes.

The cost-effectiveness analysis, from the healthcare services perspective, involves direct medical costs per quality-adjusted life year gained and two periods, a ‘within-trial’ period and a lifetime Markov model. Deterministic and probabilistic sensitivity analyses are planned.

**Discussion:**

This ongoing trial aims to set up the implementation of evidence-based programs in the clinical setting for chronic patients.

**Trial registration:**

Clinical Trial.gov NCT01657227

## Background

Type 2 diabetes mellitus (T2DM) is a paradigmatic chronic disease in which health outcomes are related to patients’ decision-making on adherence to life-style changes and pharmacologic recommendations. Besides patients, other relevant stakeholders, such as family members, as well as healthcare professionals, mainly at the primary care level, also play a relevant role in supporting patients’ decision-making.

In the Canary Islands, Spain, the prevalence of T2DM in the population over 15 years is 7.74%, slightly higher than the Spanish average (6.99%) [1]. However, the Canary Islands have an increased prevalence of diabetes-related end-stage renal disease [2-4] and diabetes-related mortality [5], when compared to the rest of Spain, with 65 vs 20–30 cases/million population and 7.8% vs 2.5%, respectively. This happens despite the fact that patients with diabetes mellitus (DM) have a mean number of ten visits/year to their primary care physician/nurse in the Canary Islands [6]. Furthermore, public healthcare resources earmarked to care for people with diabetes in the Canary Islands increased from 2.13% in 1998 to 5.9% in 2004 [7].

Despite the availability of evidence-based clinical practice guidelines [8,9] and clinical trials reporting better health outcomes linked to interventions promoting patients’ self-care [10,11], international reports still show that only 55% of people with T2DM receive diabetes education [12]; 16% adhere to recommended self-management activities [13], 37% meet the glycated hemoglobin (HbA1c) target of 7.0%, and only 7% meet combined glycemic, lipid, and blood pressure goals [14-17].

The socio-economic and public health consequences of T2DM in the Canary Islands prompted the Canary Islands Health Service (CIHS) to assess the effectiveness and efficiency of new interventions to improve both patient healthcare outcomes and the sustainability of publicly funded healthcare services. In this context, information and communication technology (ICT) offers the opportunity of efficiently supporting knowledge transfer and behavior modification interventions to improve decision-making by T2DM patients [18-22] and healthcare professionals [23-25]. Indeed, more than 70% of Canary Island families and 90% of inhabitants have daily access to internet and mobile phones, respectively [26].

Although there are many publications addressing the use of different ICT applications to support patient and professional decision-making [23,27], few studies have assessed the health and economic impact of complex interventions by means of large and long-term randomized clinical trials reporting on effectiveness and cost-effectiveness.

The Diabetes Intervention study in the Canary Islands (INDICA study) is a randomized controlled trial (RCT) that assesses the effectiveness and cost-effectiveness of three different complex interventions for knowledge transfer and behavior modification of patients, families, and healthcare professionals (physicians and nurses) at the primary care level in the Canary Islands. The interventions include a diabetes-coaching system using a combination of conventional educational workshops with mobile phones, a patient web-based platform, electronic decision aids, and periodic feedback on patients’ outcomes to guide them and healthcare professionals in decision-making related to T2DM management.

We hypothesize that this combination of conventional educational activities complemented with timely and continuous ICT decision support tools will efficiently improve disease management skills and behavior, both in patients and in healthcare professionals, in addition to health outcomes (HbA1c change over 2 years) in T2DM patients.

## Methods

### Trial design

The INDICA study is an open, community-based, multicenter, clinical controlled trial with random allocation by clusters to usual care or one of the following different interventions of knowledge transfer and behavior modification.

Group 1 corresponds to *intervention only for patients and family members*, group 2 to intervention only for *healthcare professionals at primary care*, and group 3 is a *combined intervention* for patients and professionals. In the control group, neither patients/families nor physicians/nurses receive any additional educational or supporting activities beyond the usual activities provided by the CIHS.

### Subjects

#### Patient inclusion criteria

1. Patients with T2DM diagnosed at least 1 year prior to study enrolment
2. 18–65 years of age
3. Formal consent to participate in the study
4. Regular use of mobile phone

#### Patient exclusion criteria

1. Chronic kidney disease ≥ stage 3b, as defined by the National Kidney Foundation’s Kidney Disease Outcomes and Quality Improvement Initiative (KDOQI), urinary albumin to creatinine ratio (UACR) ≥ 300 mg/g, and/or urinary protein excretion ≥ 300 mg/24 h.
2. Acute coronary syndrome (documented angina or myocardial infarction) or stroke in the last 6 months or class III or IV heart failure, according to the New York Heart Association (NYHA).
3. Proliferative diabetic retinopathy or clinically significant diabetic macular edema requiring previous treatment with retinal photocoagulation, vitrectomy, or intravitreal injections of anti-vascular endothelial growth factor or triamcinolone acetonide 6 months prior to study inclusion.
4. Uncorrected severe hearing or visual impairment or corrected visual acuity ≤ 20/40 by any cause.
5. Diabetic foot with ulcers ≥ 2 according to the Wagner scale.
6. Liver cirrhosis
7. Cancer unless disease free 5 years after diagnosis
8. Other terminal illnesses
9. Intellectual retardation, dementia, psychotic diseases
10. Active substance abuse, alcohol, or drugs (must be sober for 1 year)
11. Pregnancy
12. Insufficient (Spanish) language skills
13. Physical disability limiting participation in group education activities
14. Concurrent participation in another clinical trial or any other investigational study.

#### Primary care professionals

The unit of recruitment for primary care professionals was the Family Care Unit (FCU), composed of a family physician and a nurse. Given the interventions’ nature and the organizational characteristics in primary care at CIHS, it was agreed that physicians and nurses working together as FCUs independently sign the informed consent to participate. Family physicians and nurses either planning or awaiting placement changes among primary healthcare practices (PHCP) in the first 6 months after project initiation were excluded.

Only PHCP with at least eight FCUs and availability of appropriate places to provide group sessions were included.

### Setting and recruitment

PHCP were randomly recruited in four of the seven Canary Islands (Tenerife, Gran Canaria, Lanzarote, and La Palma). Tenerife and Gran Canaria are the main and most populated islands, providing 12 PHCP each (four from metropolitan areas, four from the south, and four from the north). La Palma and Lanzarote are less populated islands and provided four PHCP each. The Human Resources Department of the CIHS at every island supplied us with an updated list of publicly available physician/nurses for every selected PHCP. FCUs’ recruitment in PHCPs was supported by informative meetings preceded by meetings with local health authorities as well as with the directors of all selected PHCP on every island. In these meetings, a 60–80-min presentation describing the study objectives, planned time frame and tasks to be developed by healthcare professionals, expected resources utilization, and funding procedures were detailed.

After FCUs agreed and consented to participate, the electronic clinical records (ECR) of all potentially eligible patients in the selected FCU were screened to verify all inclusion and exclusion criteria. Once identified, patients received a phone call to explain the study objectives, informing that they might be eligible to participate and inviting them to an initial meeting in their respective PHCP. In this meeting, the study staff provided face-to-face extended information about the study, confirmed patient eligibility, and invited them to sign individual patient informed consent.

When a participant physician/nurse left their practice, they were excluded from the study and replaced by the new physician/nurse. In groups 2 and 3, the educational intervention was given to the new healthcare professionals on an individual level. Their corresponding patients were kept in the study without changes.

### Random assignment

Randomization was applied at different levels in every island included in the trial. Three different strata or geographic areas were set in Tenerife and Gran Canaria (metropolitan, northern, and southern areas). Four PHCP were randomly allocated to every stratum. Each PHCP was assigned to one of the three interventions or control arms by block permutation. La Palma and Lanzarote were geographically divided into four zones with only one PHCP available in each zone. Each of these PHCP was randomly assigned to one of the study arms. In every island, all arms were equally distributed.

Six FCUs were randomly selected from all those consenting participants at each PHCP. From all patients fulfilling inclusion criteria and consenting to participate in each PHCP, 15 were randomly selected per FCU. Exceptionally, more than six FCUs or more than 15 patients per FCU were selected, in order to recruit 90 patients at every PHCP.

While PHCP randomization was performed using block permutation at three different levels, second (FCUs) and third (patients) stage randomizations were performed by simple generation from a list of random numbers. PHCP assignment to interventions or control group was performed by the data manager.

In order to prevent potential contamination among study interventions, all FCUs at every PHCP were allocated to the same study group.

### Blinding

Participating FCUs were not told about their intervention assignment (groups 1–4) until the last patient agreed to participate at every FCU. To warrant patient participation and cooperation, interventions could neither be blinded to patients nor to healthcare professionals. Data analysis will be blinded to the intervention assignment.

### Interventions

**Figure 1:**
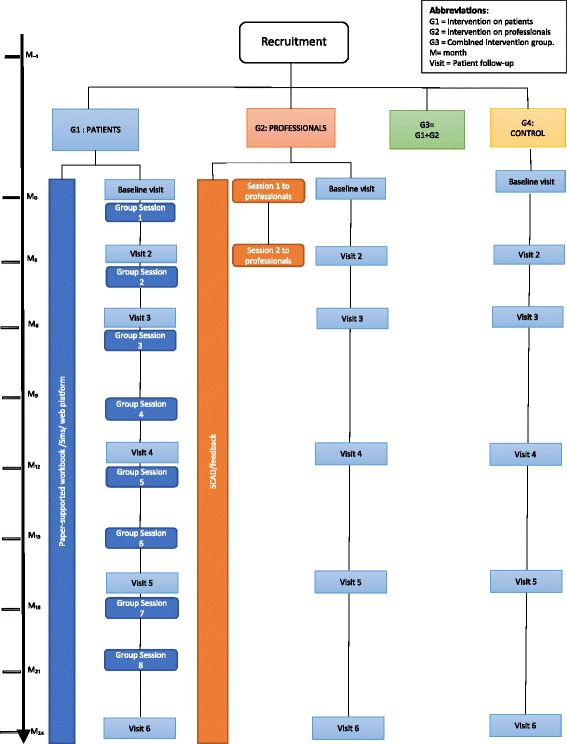
**Recruitment according to intervention assignment.**

The study will assess the effectiveness and cost-effectiveness of three different complex interventions of knowledge transfer and decision guiding for primary care healthcare professionals and/or patients and families, according to intervention assignment (Figure 1). These three interventions are compared with a control group receiving usual care.

#### Patient interventions

Groups 1 and 3 receive a complex intervention of knowledge transfer and behavior modification combining the following: A) an educational and interactive group program plus continuous monitoring by means of B) daily use of paper workbooks and weekly utilization of a web-based platform and C) tailored semi-automated mobile phone messages.

A) Interactive educational group program:
  Patients accompanied, if appropriate, by one family member responsible for buying food and cooking and serving meals are invited to receive a set of eight quarterly 3-h group sessions during the 2 years of intervention. The aim of this program is to empower patients in self-control and monitoring as well as to involve relevant family members in supporting appropriate decisions about nutritional, pharmacologic, and physical activity issues. An experienced nurse, trained in diabetes education, conducts every session, with groups of ten patients and their corresponding family members. Every session contains theoretic and practical interactively delivered activities about the most important diabetes topics: understanding T2DM, cooking, understanding nutritional food labels, glycemic target, foot care, drug adherence, tobacco, stress management, exercise, chronic complications, etc., according to Funnel et al.’s recommendations [28]. The specific contents and procedures of every session were developed based on several systematic literature reviews. The best documented and assessed educational interventions that provided valid and longer term data on improvements in relevant T2DM health outcomes were selected [29-31]. In every session, interactive activities are used to reinforce knowledge transfer and self-motivation. The first session included information and training about the adequate use of the other components of the intervention, such as the web platform, the workbooks, and the short message service (SMS) sent to mobile phones. In order to develop an educational tool for future use in research or clinical practice, these sessions are video recorded, after consent of all group participants. This recording is available on-line to patients.
B) Patient logs for continuous self-monitoring and periodic reporting:
  To provide continuous support to patients and to reinforce self-care and lifestyle changes, two different types of workbooks were developed, which show patients the dynamics and relationships of food intake, physical activity, and medication adherence with blood glucose levels. Patients are invited to use a paper-supported workbook and a web workbook embedded in a patient web-based platform. The paper version gathers daily information on the amount of physical activity, nutritional intake, medication adherence, mood, blood pressure, and blood glucose levels. In addition, this information is summarized and filled in, weekly, in the web workbook, to allow for continuous monitoring and feedback by means of automated SMS. Once the web-based questionnaire is completed, graphical feedback is displayed in the web platform, showing the variation in all selected variables over time. The web platform also offers additional information on diabetes self-management extracted from the contents of the group educational program. Additionally, every month, patients are requested to complete a longer, web-based questionnaire, collecting information on tobacco use, foot care, and weight control. This monthly questionnaire is also used to provide continuous feedback in the form of automated SMS (see below). A free phone service is available to fill in the online workbooks, as an alternative to the website access.
C) Tailored semi-automated mobile phone messages for continuous patient support:
  Mobile phones are used to warrant reception of tailored continuous support by means of semi-automatic periodic delivery of predefined SMS to support diabetes self-management about healthy diet, tobacco use, physical activity, treatment adherence, stress management, and foot care [18]. SMS contents are progressively fitted according to the topics discussed in the successive group sessions and to the degree of achievement of the different targets required to attain adequate self-management. SMS are sent weekly, focused on two different targets each week (eight targets per month). Specific SMS are selected according to a computer algorithm that reviews patient compliance for every target monthly. Compliance is classified as either ‘adequate control,’ ‘partial improvement,’ or ‘inadequate control’ for every target. The eight targets with the poorest results are selected monthly, on a two-per-week basis. Two messages a week are sent when targets attained ‘adequate control’ or ‘partial improvement.’ For targets under ‘inadequate control,’ four messages a week are activated. In addition, all patients receive one general education SMS and another as a reminder to complete questionnaires every week, up to a maximum of one SMS a day. The ICT-based interventions (components B and C of the patient intervention) were designed according to the best available evidence by means of a literature review [18,20,32,33].

#### Interventions for primary care physicians and nurses

Primary care professionals (physicians and nurses) assigned to groups 2 and 3 receive a complex intervention of knowledge transfer and decision-making composed of the following: A) an educational and interactive group program, B) continuous support by means of an automated decision aid tool embedded into the electronic clinical record of patients included, and C) periodic feedback on process and outcome measures for all T2DM patients of the corresponding FCU.

A) Educational interactive group intervention for FCUs:
  The six FCUs (physician-nurse couples) selected in every PHCP received 5 hours of education, in two interactive sessions, 3 months apart. The objectives and contents of the first session are designed to update evidence-based clinical knowledge on T2DM management, improve communication and negotiation abilities, and develop skills to promote patient-centered care [34] and shared decision-making [29]. Role-playing exercises with a set of short video-films representing different types of complex sham patients are used to deliver this intervention. The session also includes an explanation about the use of the automated decision aid tool (see below). The second session is designed to promote shared decision-making and motivational interviewing methods in the context of the patient-centered care model [29,34]. Shared decision-making is promoted throughout the sessions to help patients explore and identify their personal preferences. These sessions are led by an endocrinologist and a primary care physician with proven expertise in communication skills and patient-centered care methods [35-37]. They are video-recorded in order to standardize training and to ensure intervention reliability [38].
  The evidence-based information to update knowledge on clinical management for T2DM was obtained from the clinical practice guidelines (CPG) of the National Institute for Health and Clinical Excellence of the United Kingdom (NICE) [9] and was complemented with those developed by the American Diabetes Association (ADA) [8]. These two guidelines were selected after a process that included 1) a systematic search of guidelines in several databases (MEDLINE, PREMEDLINE, Trip Database, GuíaSalud (CPG database in Spain), and National Guideline Clearinghouse) and 2) the assessment of their quality by means of the AGREE instrument and their degree of updating [39]. Contents from these two CPG were collapsed and contextualized to obtain the INDICA CPG.
B) Decision support tool embedded in the ECR:
  Physicians and nurses have access to an automated decision support tool (DST) built by means of a computational algorithm from the previously developed INDICA CPG and integrated into the primary care ECR to adapt the recommendations to the specific needs of every patient included. This DST is passively activated, providing dynamic and interactive support for clinical management of decision-making. The tool is made available for the 15 patients included in every FCU [23-25]. As previously mentioned, the DST takes into consideration both the best available scientific knowledge [8,9] and relevant clinical information of every patient stored in the ECR (blood pressure, glucose, and cholesterol levels; renal function, comorbidities, missed tests or appointments, etc.).
C) Feedback screen:
  Every month, physicians and nurses in participating FCUs receive feedback, consisting of a computer screen displaying a personalized graphical summary of relevant processes and outcome indicators compared to mean results obtained by participating PHCP [25]. Every month, this informative screen is automatically displayed when the healthcare professional switches on his/her work PC. The screen displays combined indicators, periodically generated by automated proprietary analytical models from the ECR of all T2DM patients in the FCU and not just the 15 study participants.
  Process indicators assessed include the measurements HbA1c, blood pressure, body mass index (BMI), and lipid profile, as well as the performance of periodic screening for retinopathy and nephropathy, according to the INDICA CPG recommendations. Outcome indicators are based on the levels of HbA1c, blood pressure, BMI, and every component of the lipid profile. Outcome indicators are classified into three levels depending on whether the patients are in the expected target, not in target but better than the previous visits, or out of target and with no improvement. An overall severity indicator is also calculated by taking into consideration the number of outcome indicators out of the expected goal by patient. For every indicator displayed on the screen, mean reference values obtained from all FCUs at the same PHCP are used as dynamic comparators.

### Ethics

The Scientific and Ethics Committees of both the University Hospital of Canarias and the University Hospital Nuestra Señora de la Candelaria approved the study protocol. Moreover, a Data Safety and Monitoring Board was appointed to review and monitor the study procedures and potential adverse events. The study is being performed in accordance with Good Clinical Practice standards, applicable local regulatory requirements, and the recommendations of the Declaration of Helsinki.

### Endpoints

#### Primary endpoint

The primary endpoint of the study is the mean change in HbA1c from baseline until 24 months later. We considered a change in HbA1c of 0.4 percentage points to be clinically significant [40]. In addition to the measurements at baseline and at 24 months, HbA1c is also measured at 3, 6, 12, and 18 months (Table 1).

**Table 1:** **Outcome measurements according to periods of follow-up and type of collection**

**Time**		**Outcome measurements**
Outcomes measured on patients		
M0, M3, M6, M12, M18, M24 (F to F)		Demographic data, health history, history of DM, DM health status, current medications, risk factors for complications of poorly controlled DM
*Laboratory measurements*		
M0, M12, M24 (CT)		HbA1c; fasting glucose; total cholesterol; HDL, LDL, and non-HDL cholesterol; triglycerides; serum creatinine; albumin/creatinine ratio; and glomerular filtration rate
M3, M18 (CT)		HbA1c, fasting glucose
M6 (CT)		HbA1c, fasting glucose, total cholesterol, HDL, LDL cholesterol, and triglycerides
*Anthropometric measurements*		
M0, M3, M6, M12, M18, M24 (F to F)		BMI, waist/hip ratio, systolic and diastolic blood pressure, heart rate
*Macro and microvascular complications*		
M0, M12, M24 (F to F, ECR)		Incidence of new ischemic heart events, hospitalization for congestive heart failure, peripheral artery disease, carotid stenosis fulfilling criteria for endarterectomy or confirmed ischemic or hemorrhagic stroke, incidence or progression of diabetic retinopathy, incidence or progression of diabetic nephropathy
*Eye examination*		
M3, M24 (CT)		Retinography and macular examination by OCT
*Instruments used for self-reported outcomes measures*		
M0, M12, M24	(SRI)	ADDQoL-19, BDI-II, DES-SF, DDS2, DIATEK, IPAQ, MEDAS, STAI-S, INDICA-LSQ
	(F to F)	EQ-5D-5 L, MMAS
M6, M18	(SRI)	ADDQoL-19, IPAQ, MEDAS
	(F to F)	EQ-5D-5 L, MMAS
*Healthcare utilization*		
M0, M3, M6, M12, M18, M24 (F to F, ECR)		Visits to primary care services, nurses, specialists; hospital admissions, emergency room visits, laboratory procedures, and other diagnostic tests; medication
*Satisfaction*		
M24 (SRI)		INDICA-SATP
Outcomes measured on physicians and nurses		
M0 (F to F)		Demographic data, years in practice, practice descriptors
M0, T3 (SRI)		INDICA-KNOW, LATCon
M24 (SRI)		INDICA-SATC

BMI: body mass index; F to F: face to face interview; CT: clinical test; DM: diabetes mellitus; ECR: electronic clinical records; HDL: high-density lipoprotein; LDL: low-density lipoprotein; OCT: optical coherence tomography; SRI: self-reported interview.

Note: see description of the questionnaires in Table 2.

#### Secondary endpoints

A broad set of secondary outcomes are measured (Table 1), including the following:

- *Cardiovascular risk factors*: mean change of BMI, waist circumference, and waist-to-hip ratio, systolic and diastolic blood pressure, total cholesterol and its fractions (low-density lipoprotein (LDL), high-density lipoprotein (HDL), and nonHDL), and triglycerides. Blood pressure is measured twice in one arm (right when possible) in a sitting position, with a digital sphygmomanometer trademark OMRON© model M6, and the average of the two readings will be recorded. Smoking status is determined by self-report of whether the subject currently smoked.
- *Macrovascular diabetes complications*: new ischemic heart events (angor pectoris, myocardial infarction, surgical or percutaneous coronary revascularization), hospitalization for congestive heart failure, peripheral artery disease (surgical or percutaneous peripheral arterial revascularization, nontraumatic lower limb amputation), carotid stenosis fulfilling criteria for endarterectomy, or confirmed ischemic or hemorrhagic stroke. The annual occurrence of cardiovascular events, surgical procedures, or hospitalization is verified by reviewing the medical records.
- *Microvascular diabetes complications*: Incidence and progression of diabetic nephropathy: mean change in UACR, UACR ≥ 30 mg/g, mean change in estimated glomerular filtration rate (eGFR), eGFR < 60 mL/min/1.73 m^2^ and need for renal replacement therapy (dialysis or renal transplantation). Incidence and progression of diabetic retinopathy, according to the results of a retinography, and incidence and progression of diabetic macular edema, according to the results of an optical coherence tomography (OCT) and a retinography, are measured at months 3 and 24 of the study. Incidence of diabetic polyneuropathy is measured using the Michigan Neuropathy Screening Instrument (MNSI), an emergent instrument used to assess distal diabetic peripheral polyneuropathy. Only the 15-item self-administered MNSI, which is scored by adding up abnormal responses [41] will be applied. MNSI was translated into and back-translated from Spanish for its use in the INDICA study.
- *Health-related quality of life* (*HRQoL*), *distress*, *anxiety*, *depression*, *satisfaction with the interventions*, *health behaviors*, *and changes in knowledge about diabetes self-management*: all instruments selected to measure these outcomes are reported in Table 2.

**Table 2:** **Instruments used for self-reported outcomes measures**

**Instruments**	**Outcome measurements**
Outcomes measured on patients	
EQ-5D-5L [59]	Generic HRQoL questionnaire. The self-reported description assesses five domains: mobility, self-care, usual activity, pain/discomfort, and anxiety/depression
ADDQoL-19, Audit of Diabetes-Dependent Quality of life [60]	Specific HRQoL questionnaire for DM. It assesses 19 domains: leisure activities, working life, travel, holiday, physical activities, family life, social life, personal life, sex life, physical appearance, self-confidence, motivation, reaction from others, feelings about the future, financial situation, living conditions, reliance on others, freedom to eat, and freedom to drink
DDS2, Diabetes Distress Scale [61]	It is a validated two-item diabetes distress-screening instrument that asks respondents to rate on a six-point scale the degree of distress caused by the two following items: (1) feeling overwhelmed by the demands of living with diabetes and (2) feeling that I am often failing with my diabetes regimen
STAI-S, State Trait Anxiety Inventory [62]	It is a self-description questionnaire including two non-dependent scales, the applied state-anxiety scale (STAI State) and the trait-anxiety scale (STAI Trait). It assesses transient emotional state or condition as characterized by subjective feelings of tension and apprehension that can fluctuate in time and intensity
BDI-II, the Beck Depression Inventory II [63]	It is a validated 21-item self-report inventory that measures depressive symptoms such as sadness, pessimism, suicidal thoughts or wishes, tiredness or fatigue, loss of energy, and loss of pleasure, among others
DES-SF, Diabetes Empowerment Scale-Short Form [64]	This questionnaire assesses patient empowerment on T2DM management, including eight items with responses on a five-point Likert scale
IPAQ, International Physical Activity Questionnaire. [65]	This questionnaire checks physical activity and provides information on the time spent on walking, moderate-intensity activities, and vigorous and sedentary activities
MEDAS, Mediterranean Diet Adherence Screener [66]	This questionnaire assesses diet recommendation adherence. It consists of 14 targets for food consumption rated with one point for each target achieved
MMAS, Morisky Medication Adherence Scale [67]	This questionnaire assesses the medication adherence, including a four-item self-report measure with an established concurrent and predictive validity
INDICA-SATP	Patient satisfaction and usability of the web portal and the mobile phone communication system are assessed with a specific instrument created in the context of this project
Diatek	It is a specific instrument created in the context of this project, to assess potential changes in patient knowledge about DM based on the CPG INDICA
INDICA-LSQ	It is a specific instrument created in the context of this project used to assess attitudinal changes of patients regarding lifestyles, based in the Transtheoretic Model of Behavior Change [68]
Outcomes measured on physicians and nurses	
LATCon, Leeds Attitude toward Concordance scale [69]	It is a 12-item self-reported scale to assess patients’ and health professionals’ attitudes toward concordance in medicine-taking
INDICA-KNOW	Knowledge change among healthcare professionals will be measured with the aid of an instrument with 20 questions based on the contents of the INDICA CPG
INDICA-SATC	Acceptability and usability of the DST and the feedback screen is measured according to four different dimensions: acceptability of interactions and time devoted using the software communication technology, impact on patients, impact on the clinician’s practice, and communications issues such as quality of feedback and formats used [70]

DST: decision support tool.
- *Attitude toward concordance and knowledge about the clinical management of diabetes*: these instruments, reported in Table 2, are used to evaluate the interventions on physicians and nurses included in groups 2 and 3.

##### Healthcare utilization

Costs because of the clinical management of T2DM in all groups will be assessed from the healthcare services perspective, including the costs related to the development and use of all components for each intervention assessed (group sessions, ICT system, SMS services, computer-assisted aids, etc.). The analysis will also include costs because of patient contacts with primary care services, hospital admissions and length of stay, outpatient visits, emergency attendances, and prescribed medications. The volume of resource used for each cost component will be measured with the aid of patient questionnaires and ECR; unit costs will be taken from standard published sources when available and from the specific providers.

#### Measurement procedures

Information needed from patients are being collected by several procedures, including face-to-face interviews, clinical examinations, analysis of data stored in the ECR, downloading of information from the INDICA web platform, and self-completed questionnaires. Results of laboratory tests will be downloaded from ECR by trained staff blinded to patient group assignment.

Information needed from FCUs is being obtained by means of personal interviews and self-reported questionnaires.

The planning of information collection for every outcome measured throughout the project is shown in Table 1.

#### Biochemical determinations

Blood and urine samples are being collected after an overnight fast, by research nurses, using the available facilities of the participating PHCPs. After centrifugation, samples are being immediately transported to the biochemistry laboratory of the corresponding reference hospital. HbA1c is being quantified according to the Diabetes Control and Complications Trial assay. LDL cholesterol will be estimated using the Friedewald formula, and nonHDL cholesterol will be calculated as the difference between total cholesterol and HDL cholesterol. Estimated glomerular filtration rate will be calculated using the Modification of Diet of Renal Disease formula (MDRD4).

#### Biological samples for future research questions

To facilitate efficient answers to potential future research questions on T2DM in the Canarian population, urine and blood samples of patients are being frozen and stored. This will enable collating a collection of biological specimens from a wide and representative sample of the overall T2DM population in the Canary Islands. Urine and serum samples are being obtained at baseline and 24 months, while DNA samples are only being obtained at baseline. Every patient will be specifically informed and asked to consent to storage of DNA and nonDNA biological samples. These samples are being stored in the biobanks of the hospitals of the Canary Islands belonging to the Spanish National Biobanks Network. This process is in accordance with prevailing Spanish laws on protection of personal data [42], patient autonomy [43], and biomedical research [44]. These materials will subsequently be used to search for genetic or biological markers that could either characterize the T2DM patient population or predict clinical disease course.

#### Statistical methods

Generalized linear mixed models with repeated time measurements will be used to analyze changes in outcomes over time. To compare the three interventions and the control group after different follow-up periods (Baseline, 3, 6, 12, 18, and 24 months), the intervention groups will be treated as a ‘factor within.’ First, we will examine whether the intervention in Groups 1 and 2 are better than usual care, and then, we will examine whether the most intensive intervention (group 3) is better than less intensive interventions (groups 1 and 2). The purpose of these analyses is to obtain preliminary estimates regarding the incremental benefits of the intervention components [45]. For multiple comparisons, the *P* value will be adjusted with Bonferroni correction (Pc Z corrected value). In addition, the models will include a subset of covariates that are imbalanced at baseline. To identify the covariates to be included in the model, we will first fit separate models including each covariate, one at a time. The final model will include those covariates such that their inclusion changes the estimates’ treatment effect by at least 10%. As suggested in the CONSORT statement, decisions about covariates will not be based on *P* value [46,47].

To incorporate the effect of cluster analysis, a multilevel model (MLM) approach will be implemented. MLM adjusts for the clustering effects across three levels (patients, FCU, and PHCP) of the hierarchical data structure.

For the main intention-to-treat analysis comparing outcomes, all patients will be included. Standard imputation methods (i.e., mean value imputation, last observation carried forward) will be used to impute missing data depending on the pattern of missing data. All tests will be two-sided with a type I error of 5%. Statistical analyses will be performed using Statistical Package for social Sciences (SPSS v.21, Chicago, IL, USA).

##### Sample size calculation

We estimated that 393 patients per arm (total in the study = 1,572) were needed to detect an absolute difference in Hba1c of 0.4%, assuming a common standard deviation of 1.4% [40], a two-tailed power of 90%, and an alpha of 0.05. After an additional adjustment for clustering of patients within FCU by the design effect [48], assuming 15 patients per FCU and an intra-class correlation coefficient of 0.01 (interquartile range: 0 to 0.032) based on data from the literature [49], the estimated number of patients per arm was 448 (total in the study = 1,792). Although the unit of allocation was the PHCPs, these are formed of several healthcare centers throughout the territory that only share administrative management and some services. Also, this effect was already controlled by means of the stratification. Therefore, we considered that the intra-class correlation within PHCP was insignificant, and we used instead the intra-class correlation for FCU, that is, among patients served by the same FCU. Although small, we consider that this correlation is significant.

However, sample size was increased by an additional 30% to accommodate for expected losses to follow-up and to warrant the presence of each arm in the study in the different islands. Hence, we aimed for a total sample size of 2,330.

#### Cost-effectiveness analysis

We will undertake a detailed analysis of the cost and the cost-effectiveness of each of the four groups in comparison to the others. Our analysis will conform to accepted economic evaluation methods. We will estimate cost and cost-effectiveness for the ‘within-trial’ period (2 years/short-run model) and also over the expected lifetime of the patient (lifetime/long-run model).

##### Short-run model

The cost-effectiveness measures in the two-year model will be the incremental cost per quality-adjusted life year (QALY) gained. QALYs will be calculated based on the HRQoL data collected during the trial. HRQoL will be measured according to the EQ-5D-5 L, which will be collected at baseline and at each follow-up visit for each individual patient. Patient-specific utility profiles will be constructed assuming a straight line relation between each of the patients’ EQ-5D-5 L scores at each follow-up point. The QALYs experienced by each patient from baseline to 2 years will be calculated as the area underneath this profile. We will investigate differences in baseline characteristics and, if necessary, use regression methods to control for them. As explained above, costs included in the analysis are those incurred by the healthcare service. Cost-effectiveness will be calculated as the incremental cost-effectiveness ratio (ICER) by dividing the estimated differences in costs by the differences in effects observed. Nonparametric methods to calculate confidence intervals around the ICER based on bootstrapped estimates of the mean cost and effect differences will be used. The bootstrap replications will also be used to construct a cost-effectiveness acceptability curve, which will reveal the probability that each alternative is cost-effective at 2 years for different values of willingness to pay for an additional unit of effectiveness. We will also subject the results to extensive deterministic (one-, two-, and multiway) sensitivity analysis.

##### Long-run model

The interventions under evaluation in this study are likely to have an impact beyond the trial period. To capture these potential effects, we will extrapolate the results to an extended time horizon in the analysis, i.e., considering the remaining life expectancy of the patients.

We will consider the potential application of the Centers for Disease Control-Research Triangle Institute (CDC-RTI) Diabetes Cost-Effectiveness Model [50] to estimate long-term outcomes in our population. The CDC-RTI Diabetes Cost-Effectiveness Model is a validated simulation model of disease progression and cost-effectiveness for T2DM based on data from the UK Prospective Diabetes Study (UKPDS) [51] and other sources. The aim of this model is to simulate the development of T2DM-related complications on three microvascular disease paths (nephropathy, neuropathy, and retinopathy) and two macrovascular disease paths (coronary heart disease and stroke). The model structure is based on a Markov model which simulates the progression of a patient based on estimated transition probabilities between possible disease states. In the CDC-RTI Diabetes Cost-Effectiveness Model, transition probabilities depend on risk factors—including HbA1c and cholesterol concentrations.

Following decisions about model structure to estimate future outcomes, a list of parameter estimates required for the model will be developed. Data from the trial will be used to input the model in order to estimate the long-term cost-effectiveness of the different alternatives, alongside relevant data from the published literature. The specific details of the data to be used to populate the model will be determined following the development of the structure and the systematic literature searches to identify available evidence. The cost-effectiveness measure will again be expressed in terms of the ICER for each alternative after discarding dominated strategies. We will undertake deterministic (one-, two-, and multiway) and probabilistic sensitivity analysis, the latter assuming appropriate distributions and parameter values [52].

#### Duration of fieldwork

Fieldwork is estimated to last 3 years. The first year to complete recruitment of patients and healthcare providers in primary care and the following 2 years for follow-up and measurement. As interventions are maintained over time, the period of intervention and follow-up overlap (Figure 1).

#### Monitoring

Trial monitoring is the responsibility of a research team in charge of all quality control activities, assessing adherence to the trial protocol: timely work plan execution and comprehensiveness of data acquisition and data quality (databases have been designed to avoid downloading inappropriate values for every variable). The interactive group sessions for patients and family members, as well as those for primary care physicians and nurses, are being recorded to monitor the quality of the intervention and its adherence to the predefined script.

### Trial status

Recruitment is complete and the trial is ongoing.

## Discussion

The ongoing INDICA study is a four-arm RCT involving all main actors playing a role in decision-making in T2DM (patients, families, physician and nurses). The INDICA study will assess the comparative effectiveness and cost-effectiveness of usual care for T2DM patients against three multicomponent education and coaching interventions. These interventions combine conventional group educational and training activities with different ICT-based interventions to guide the decisions of T2DM patients, families, and primary care healthcare professionals, according to evidence-based guidelines. The primary analysis is aimed at comparing the mean 24-month HbA1c (operationalized as HbA1c % change from baseline) among patients with T2DM whose PHCPs were assigned to the usual care group with the mean 24-month HbA1c % change among patients with T2DM whose PHCPs were assigned to the three different intervention groups. The study used cluster randomization to reduce the risk of contamination bias, since the educational parts of the multicomponent interventions for patients and healthcare professionals were applied to groups.

The importance of glucose control in T2DM has been confirmed in a meta-analysis [53]. The high incidence of macrovascular complications, such as myocardial infarction, stroke, and lower-limb amputations, are a major cause of disability, mortality, and economic losses. Microvascular complications, including retinopathy, neuropathy, and kidney disease also account for a highly significant morbidity, mortality, and economic burden [54] among patients with T2DM. The incidence of these complications and their healthcare and social and economic consequences is higher in the Canary Islands than in the rest of Spain and most western countries [2-4].

The interventions assessed by the INDICA study are based on the conceptual framework of behavioral change and patient-centered care [29,34]. There is increasing evidence that good self-care is related to improved T2DM outcomes [10,11,55,56]. Provider education and continuous feedback to patients and the use of reminders have been associated with improvements in provider adherence to guidelines and with clinically significant improvements in patient outcomes [10,11,55,56]. Although ICT-based studies to improve diabetes self-management have grown rapidly, there is a substantial discrepancy between the demand for this healthcare delivery mode and the scientific evidence supporting its efficacy and cost-effectiveness. Most published studies are focused on single interventions exclusively aimed at patients and are limited by methodologic deficiencies related to small-sample sizes and inconsistent selection of outcomes and measurement instruments, as well as short follow-up periods [57]. Although several studies assessing the effectiveness of ICT-based interventions on diabetes outcomes have reported small but significant effect-sizes [18-22,58], very few have assessed cost-effectiveness. Cost-effectiveness is especially relevant for the assessment of ICT-based interventions aimed at prevalent chronic diseases, given that the highest costs of the interventions correspond to the development of ICT applications whose effectiveness will become blurred over time as well as with their use by thousands of patients and physicians. In the current times of financial crisis, interventions not only have to prove effectiveness but also cost-effectiveness to reduce uncertainty in healthcare policy decision-making to contribute to the economic sustainability of public healthcare services. Consequently, while much is promised by electronic communications and tele-health interventions, there is a lack of robust information to support decisions at patient, clinician, and healthcare policy decision-maker level.

The current worldwide availability of mobile phones and internet use across socio-economic, gender, and age groups, combined with their unique ability to process and communicate data in real-time, make them an ideal platform to create simple, effective, and real-time diabetes management programs that can be used for large groups of patients. Few previous studies of electronic communication interventions for T2DM are randomized, include a control group, or involve more than one treatment group to evaluate complex or multicomponent interventions for all actors involved, not only from the effectiveness perspective but also the assessment of cost-effectiveness. This approach will improve transferability by extending the usefulness of the expected results beyond patients and clinicians in primary care to healthcare policy decision-makers.

## Abbreviations

BMI: Body mass index
CIHS: Canary Islands Health Service
CPG: Clinical practice guidelines
CT: Clinical test
DM: Diabetes mellitus
DST: Decision support tool
eGFR: Estimated glomerular filtration rate
ECR: Electronic clinical records
F to F: Face-to-face interview
FCU: Family Care Unit
HbA1c: Glycated hemoglobin
HRQoL: Health-related quality of life
ICER: Incremental cost-effectiveness ratio
ICT: Information and communication technology
M: Month
OCT: Optical coherence tomography
PHCP: Primary healthcare practices
QALY: Quality-adjusted life year
RCT: Randomized controlled trial
SMS: Short message service
SRI: Self-reported interview
T2DM: Type 2 diabetes mellitus
